# Intraguild dynamics of understudied carnivores in a human‐altered landscape

**DOI:** 10.1002/ece3.6290

**Published:** 2020-04-27

**Authors:** Tara Easter, Paola Bouley, Neil Carter

**Affiliations:** ^1^ School for Environment and Sustainability University of Michigan Ann Arbor MI USA; ^2^ Gorongosa National Park Chitengo Mozambique

**Keywords:** competition, conservation, niche partitioning, species interactions

## Abstract

Mesocarnivores constitute a diverse and often abundant group of species, which are increasingly occupying hweigher trophic levels within multi‐use landscapes. Yet, we know relatively little about their interactions with each other, especially in human‐altered areas. Using camera trap data collected in a forestry concession in the Greater Gorongosa ecosystem of central Mozambique, we examined the spatiotemporal relationships and potential for intraguild competition among three understudied African carnivores: African civets (*Civettictis civetta*), bushy‐tailed mongooses (*Bdeogale crassicauda*), and large‐spotted genets (*Genetta maculata*). After accounting for habitat preferences and tolerance to anthropogenic factors, we found that African civets and bushy‐tailed mongooses avoid each other spatially and temporally. Additionally, civets and mongooses were also both more likely to use sites farther away from human settlements, possibly decreasing the total available habitat for each species if competition is driving this spatial partitioning. In contrast, we did not find evidence for spatial or temporal partitioning between large‐spotted genets and African civets, but bushy‐tailed mongooses altered their activity patterns where they co‐occurred with genets. Our study contributes to scant ecological knowledge of these mesocarnivores and adds to our understanding of community dynamics in human‐altered ecosystems.

## Graphical abstract

We examined the spatiotemporal relationships and potential for intraguild competition among three understudied small carnivores: African civets (*Civettictis civetta*), bushy‐tailed mongoose (*Bdeogale crassicauda*), and large‐spotted genets (*Genetta maculata*). We found that civets and mongoose avoid each other, while genets occupied the same, fine‐scale spatial and temporal niches as civets and mongooses. Such interspecific interactions are important to consider for conservation planning because this information allows for better prediction of how trophic networks might shift and function in anthropogenic landscapes, which prevail globally.

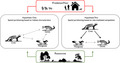

## 1 INTRODUCTION

Human population growth has caused rapid land use changes and the decline of apex carnivore populations (Ripple et al., 2014). As a result, small‐ to midsized carnivores (<15 kg, mesocarnivores hereafter; Roemer, Gompper, & Valkenburgh, 2009) are more frequently occupying higher trophic levels than in the past, altering ecosystem dynamics (i.e., mesocarnivore release; Crooks & Soulé, 1999; Ritchie & Johnson, 2009). Spatial and temporal activity patterns of mesocarnivores are typically shaped by habitat and food preferences and interactions with dominant species (e.g., Rich, Miller, Robinson, McNutt, & Kelly, 2017). In multi‐use areas, mesocarnivores must also navigate human‐altered landscapes and human activities. People have had profound impacts on entire animal communities through the exploitation of species, influencing landscapes of fear (Berger, 2007), changing the physical environment (Ellis, 2011), and fundamentally changing how species interact with each other (Moll et al., 2018; Oriol‐Cotterill, Macdonald, Valeix, Ekwanga, & Frank, 2015). Indeed, reducing risks of encounters with humans likely plays a major role in where mesocarnivores and their prey distribute themselves across the landscape (i.e., landscape of fear; Gaynor, Brown, Middleton, Power, & Brashares, 2019). This in turn might cause sympatric mesocarnivore species to have fewer opportunities to partition in space and time (Kiffner, Wenner, LaViolet, Yeh, & Kioko, 2015; Moll et al., 2018; Rota et al., 2016). Alternatively, species more tolerant of anthropogenic landscapes and activity may use these areas as refuges from their competitors (i.e., the “human shield” hypothesis; Berger, 2007). Resource acquisition, competitor avoidance, and human avoidance or tolerance collectively determine the activity patterns of mesocarnivores, driving spatial and temporal niche partitioning and altering the ecosystem services they provide (Schuette, Wagner, Wagner, & Creel, 2013; Smith, Thomas, Levi, Wang, & Wilmers, 2018; Wang, Allen, & Wilmers, 2015; Williams et al., 2017).

Dynamics among mesocarnivores and their resulting effects on community composition and trophic cascades have been well studied in North America, Europe, and Australia (e.g., Johnson & VanDerWal, 2009; Levi & Wilmers, 2012; Pasanen‐Mortensen, Pyykönen, & Elmhagen, 2013; Sivy, Pozzanghera, Grace, & Prugh, 2017) and for larger carnivores in Africa (e.g., Creel & Creel, 1996; Durant, 1998; Rich et al., 2017). For example, in the absence of wolves (*Canis lupus*) in much of the United States, coyotes (*Canis latrans*) have become dominant carnivores, suppressing or changing the activity patterns of smaller carnivores such as foxes (*Urocyon cinereoargenteus*, *Vulpes velox*, and *Vulpes vulpes*) and increasing bird diversity (Fedriani, Fuller, Sauvajot, & York, 2000; Harrison, Bissonette, & Sherburne, 1989; Levi & Wilmers, 2012; Thompson & Gese, 2007). In urban environments, these dynamics change such that foxes, which are more tolerant of human infrastructure, more frequently use urban areas as a refuge from coyotes while still avoiding people spatially and temporally (Moll et al., 2018).

Despite the abundance and diversity of mesocarnivores in sub‐Saharan Africa, however, little is known about their intraguild dynamics. African civets (*Civettictis civetta*) and large‐spotted genets (*Genetta maculata*), for example, are widespread, and their diet and habitat preferences overlap with several other carnivores (Caro & Stoner, 2003). This theoretically makes them vulnerable to interspecific competition (Caro & Stoner, 2003), yet little is known about their spatial or temporal activity patterns or how they interact with each other (Admasu, Thirgood, Bele, & Laurenson, 2004; Do Linh San et al., 2013; Ramesh & Downs, 2014). Of the studies that have investigated mesocarnivore interactions (e.g., Maddock & Perrin, 1993; Ramesh, Kalle, & Downs, 2017; Rich et al., 2017; de Satgé, Teichman, & Cristescu, 2017; Schuette et al., 2013; Waser, 1980), few have occurred outside of protected areas or have incorporated human activities into their models. How mesocarnivores partition spatially and temporally to avoid each other may shift in human‐modified landscapes, depending on their tolerance for human presence and activities. Furthermore, some of these studies only investigate co‐occurrence among potentially competing mesocarnivores without incorporating habitat preferences (Ramesh et al., 2017; de Satgé et al., 2017) or anthropogenic factors (Rich et al., 2017), which may be stronger drivers of spatial or temporal activity patterns than the potential competitor. The paucity of ecological data on the mesocarnivores of sub‐Saharan Africa, the high potential for competitive interactions between them, and their shifting ecological roles in multi‐use landscapes highlight important knowledge gaps. To help fill these knowledge gaps, we used camera trap data to better understand the spatiotemporal dynamics of mesocarnivore site use in a forestry concession in the Greater Gorongosa ecosystem of central Mozambique. The Gorongosa ecosystem could provide an interesting case study on how mesocarnivores interact with each other in human‐modified landscapes, due to the low densities of large carnivores (e.g., lions (*Panthera leo*), leopards (*Panthera pardus*), hyenas (*Crocuta, Hyaena brunnea*), and wild dogs (*Lycaon pictus*)) following decades of civil unrest in the region, and growing human populations and infrastructure development (Bouley, Poulos, Branco, & Carter, 2018; Easter, Bouley, & Carter, 2019). Here, mesocarnivores face relatively few top‐down pressures aside from potentially competing among each other and avoiding people. This allows us to test theories about interspecific competition among species with shared ranges, habitats, diets, and body sizes (Maddock & Perrin, 1993; de Satgé et al., 2017). For example, temporal overlap among activity patterns of competing carnivores could facilitate spatial partitioning among them (Carter, Jasny, Gurung, & Liu, 2015). Alternatively, subordinate species may have a higher temporal overlap with people if dominant competitors displace them from more preferred time periods (Schuette et al., 2013).

**FIGURE 1:**
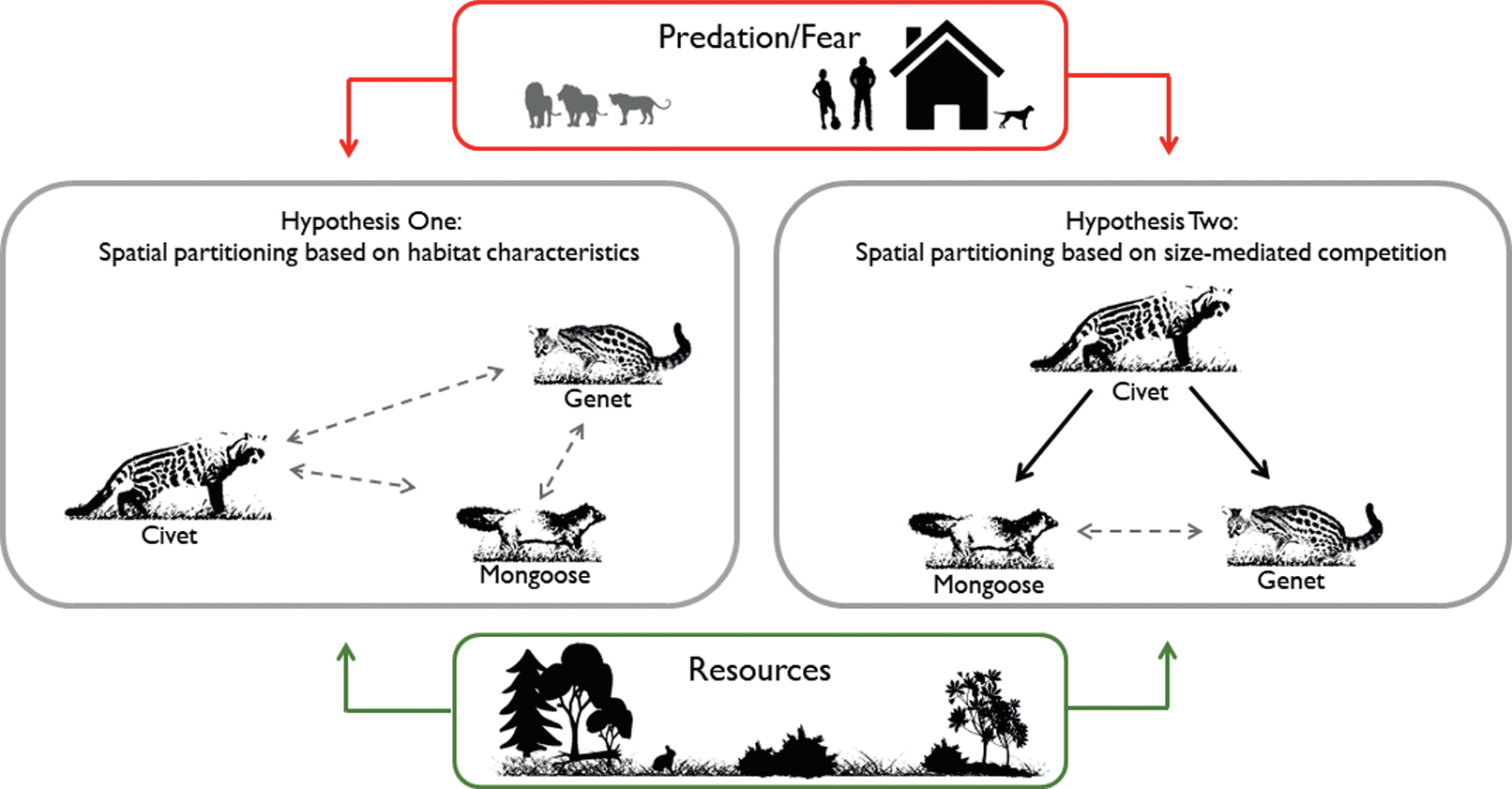
Conceptual diagram of hypothesized spatial relationships between African civets, large‐spotted genets, and bushy‐tailed mongooses, accounting for the influences of top‐down (Predation/Fear) and bottom‐up (Resources) factors. In Hypothesis 1, these three species have little relative influence on each other (dashed arrows) and are spatially distributed based on habitat preferences. In Hypothesis 2, spatial distributions are largely determined by competitive interactions based on body size (solid arrows)

We investigated the potential for competitive interactions among three common but understudied mesocarnivores: African civets, large‐spotted genets, and bushy‐tailed mongooses (*Bdeogale crassicauda*; Table 1). We tested two hypotheses (Figure 1). Our first hypothesis is that these species will segregate in space based largely on habitat preferences and tolerance of people. Several studies have shown that genets are more tolerant of areas with people than other carnivores (Fuller, Biknevicius, & Kat, 1990; Pettorelli, Lobora, Msuha, Foley, & Durant, 2010; Ramesh & Downs, 2014; Schuette et al., 2013), and bushy‐tailed mongooses prefer forested areas (Caro & Stoner, 2003; Kingdon, 2015; Pettorelli et al., 2010). Our second hypothesis is that the smaller mesocarnivores (genets and mongooses) will avoid the larger mesocarnivore (civets) in space and/or time due to being at a competitive disadvantage for resources. Body size can influence competitive interactions among species, with larger species able to outcompete or directly harm smaller species (Donadio & Buskirk, 2006; Palomares & Caro, 1999). For example, de Satgé et al. (2017) found that striped polecats (*Ictonyx striatus*) and small‐spotted genets (*Genetta genetta*) avoided their larger competitor, the African wildcat (*Felis silvestris lybica*), but these relationships have not been examined for our study species. Species interactions shape community structure, abundance, and distributions, and may have important cascading effects on ecosystem services and function (Crooks & Soulé, 1999; Schuette et al., 2013; Williams et al., 2017). Understanding intraguild interactions among species in varying environmental conditions (e.g., low competition risk from large carnivores, varying degrees of anthropogenic disturbance) allows conservation managers to better predict the species of mesocarnivores that are most vulnerable to anthropogenic changes, assess the indirect effects on other species in the community, and weigh the risks to wildlife populations while managing landscapes for human and wildlife coexistence (Cardillo et al., 2005; Pettorelli et al., 2010).

**TABLE 1:** Ecological characteristics of our three study species observed in central Mozambique: African civets, bushy‐tailed mongooses, and large‐spotted genets

Common name	Species name	Size	Range	Home range size	Habitat preferences	References
African civet	*Civettictis civetta*	7–20 kg	Widely distributed in sub‐Saharan Africa	5–11 km^2^	Anywhere with adequate cover and food, usually near water.	Caro and Stoner (2003) (size, habitat); Estes (2012) (range, home range, habitat)
Bushy‐tailed mongoose	*Bdeogale crassicauda*	2 kg	Common within portions of Tanzania, Mozambique, Zimbabwe, Zambia, and Malawi	Unknown	Woodland/scrub and forested areas.	Caro and Stoner (2003) (size, habitat); Pettorelli et al. (2010) (range); Rovero et al. (2017) (habitat)
Large‐spotted genet	*Genetta maculata*	2 kg	Widely distributed in sub‐Saharan Africa	0.5–1 km^2^	Anywhere with adequate cover and food. Tolerant of human‐modified areas.	Caro and Stoner (2003) (size, habitat); Estes (2012) (range, home range); Schuette et al. (2013) (habitat)

All species are nocturnal and opportunistic generalists. However, these species consume varying amounts of small vertebrates, invertebrates, fruits, and plants which may affect spatial and temporal partitioning.

## 2 MATERIALS AND METHODS

### 2.1 Study area

**FIGURE 2:**
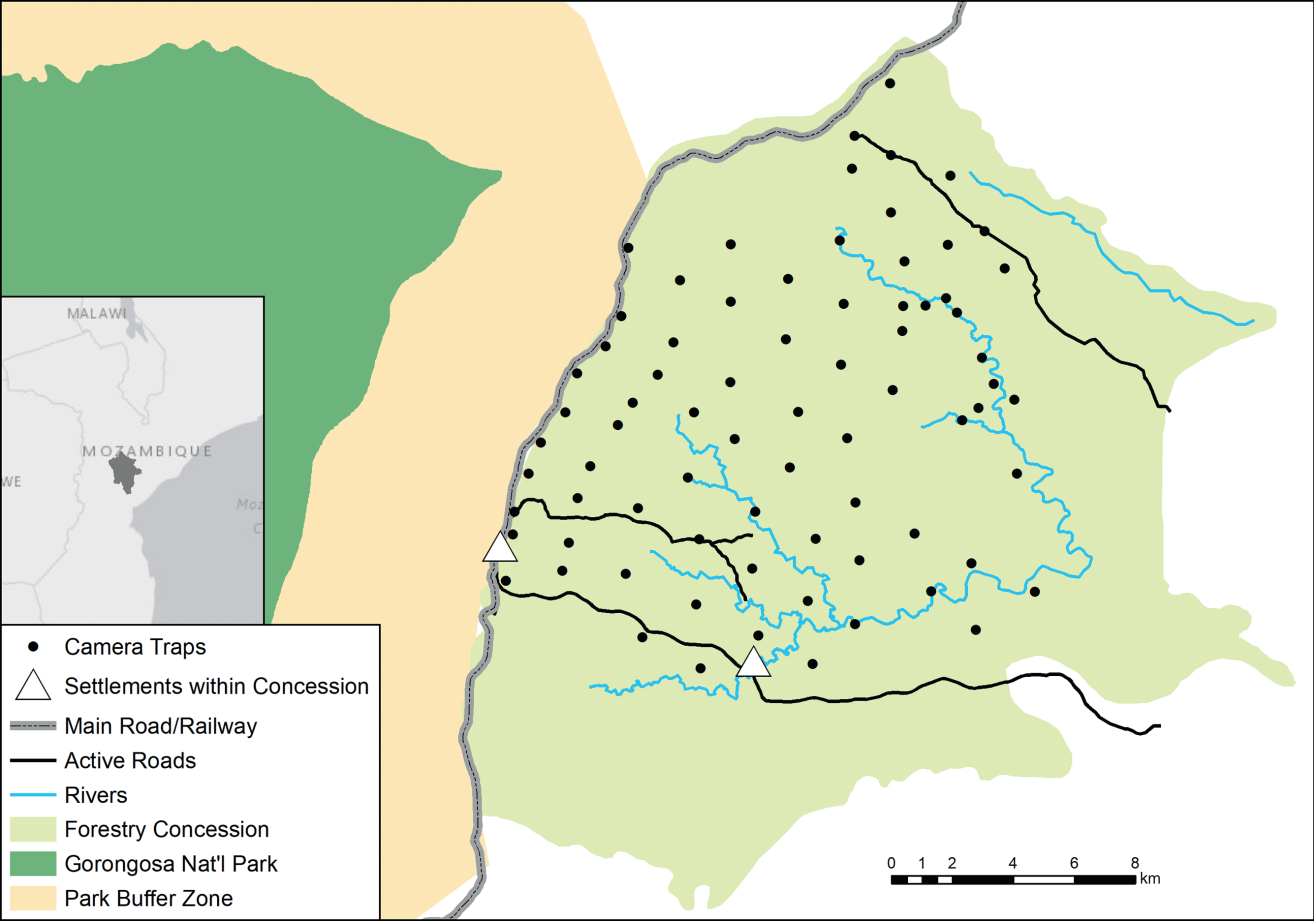
Map of our study site within a sustainable forestry concession adjacent to Gorongosa National Park and its buffer zone in central Mozambique

Our study site was in central Mozambique, east of Gorongosa National Park's buffer zone. This area has a subtropical climate with a wet season from November to April and a dry season from May to October. We conducted our surveys in a Forest Stewardship Council (FSC)‐certified forestry concession (460 km^2^; Figure 2) composed mostly of miombo woodlands (*Brachystegia* spp.) with a range of tree cover from patches of dry miombo woodlands and open grasslands to moist, closed‐canopy riverine forests (Stalmans & Beilfuss, 2008). Elevation decreases gradually from approximately 350 to 150 m from the Cheringoma Plateau in the west to the confluence of the Chiteme and Chimiziua rivers to the east. There are two small settlements (<500 households) within the concession: Condue to the southwest and the forestry's sawmill and living headquarters in the southeast. All roads in the concession are single‐track, dirt roads, created mainly for timber harvest, and a larger road and parallel railway bisects the concession and the park's buffer zone. Roads that were not being used for concession activities were mostly inactive and grown‐over. Our team only conclusively documented two individual leopards and no other large carnivores (e.g., hyena, wild dog, lion) at the time of this study. This allowed for studying how mesocarnivores interact with minimal influence of larger, dominant carnivores in the area.

### 2.2 Carnivore detection data

To measure carnivore site use, we deployed infrared camera traps (Bushnell Trophy Cam 24MP and 14MP no‐glow Aggressors) at 77 sites within the forestry concession. We used a 4 km^2^ hexagonal grid with approximately 2 km separating each site to guide our camera trap placement, but we prioritized roads and animal trails where possible, following protocols from other studies that quantified carnivore space use (Carter, Shrestha, Karki, Pradhan, & Liu, 2012; Rosenblatt et al., 2016). Due to a limited number of cameras and time for deployment, traps consisted of either pairs or single cameras to protect against possible failures while covering greater areas, and we rotated traps in four successive blocks from June to October 2017 (Ahumada, Hurtado, & Lizcano, 2013; Rovero et al., 2017; Sollmann, Gardner, & Belant, 2012). Each camera trap was active for an average of 28 days (Athreya, Odden, Linnell, Krishnaswamy, & Karanth, 2013; Wegge, Odden, Pokharel, & Storaas, 2009). We mounted each camera on a tree at about 45–60 cm above the area or trail of interest. Identifying individuals with these cameras, especially at traps with only one camera, is challenging. To reduce detection bias, we only considered detections of the same species independent if they occurred at least 30 min after the last time that species was detected at that trap, regardless of if another species passed within those 30 min (O’Connor et al., 2017; Wang et al., 2015).

### 2.3 Temporal overlap

To investigate the interactions between mesocarnivores, we first examined their daily activity patterns for temporal overlap. Each species is considered nocturnal (Estes, 2012; Pettorelli et al., 2010), but fine‐scale avoidance between species could lead to temporal niche partitioning (Carter et al., 2015; Schuette et al., 2013). We extracted the time stamps from each independent photo of bushy‐tailed mongooses, civets, and genets to create kernel density estimates of daily activity patterns of each species. We compared the activity patterns of each species from camera traps it was detected without a potential competitor to activity patterns of that species at camera traps where it and its potential competitor were detected. These density distributions were used to calculate the coefficient of overlapping, $\hat{D}$ , which ranges from 0 to 1, with 1 representing complete temporal overlap between the estimated activity times of a species pair, and 0 representing no temporal overlap between a species pair. We report $\hat{D_{1}}$ due to smaller sample sizes in some comparisons (fewer than 75 observations) and consider $\hat{D_{1}}>0.80$ (approximately) to be a strong overlap (Allen, Peterson, & Krofel, 2018). We performed all analyses in R (R Core Team, 2013), using the package “overlap” (Meredith & Ridout, 2017).

### 2.4 Co‐abundance

#### 2.4.1 Analysis

We used two‐species, N‐mixture models to estimate the abundance of mesocarnivores relative to each other while accounting for differential environmental effects and imperfect detection (Brodie et al., 2018; Royle, 2004). Because we did not identify individuals, a site where 20 mongooses, for example, were detected could be 20 detections of the same individual repeatedly using that site in front of the camera. Therefore, we refer to the predicted abundances produced by these models as a metric for how often a species used a given site. N‐mixture models use repeated counts of a population over time to estimate local abundance for a species *i* at location *j* (*N_i,j_*) by assuming *N_i,j_* ~ Poisson (λ*_i,j_*). The number of independent detections of a species in one day was counted as one count. Thus, if a camera trap was active for 20 days, there were 20 counts. We modeled the expected count of a species *i* at each location *j* (λ*_i,j_*) given environmental and anthropogenic covariates using a log‐link function (Royle, 2004). To include the effect of one species’ abundance on another, δ estimates the coefficient, or effect, of a species’ abundance (*N*_1_) on the other species in a pair: log (λ_2,_ *_j_*) = *α*_2_ + *α*_2_ (Covariate)*_j_* … + δ*N_1,_ *_j_*.

An estimated negative value of δ would therefore indicate a negative correlation between the abundances of species 1 and species 2, suggesting the potential of competitive exclusion (Brodie et al., 2018). A positive estimate indicates that abundances of the two species increase together, which could indicate a lack of competitive effects (Brodie et al., 2018), optimal habitat and sufficient resources for both species (Rich et al., 2017), or, in some cases, mutualistic relationships. We considered δ estimates significant if the 95% credibility interval (CI) did not overlap zero. Similar to other occupancy models (MacKenzie et al., 2002; Mackenzie & Royle, 2005), N‐mixture models assume population closure.

The strength of this modeling approach lies also within its ability to account for imperfect detection and mitigate biases that may alter estimations of *N_i,j_*, as true abundance cannot be observed. To do this, the species‐level detection probability (*p*) is modeled as *p_i,j,k_*: *n_i,j,k_* ~ Bin (*N_i,j,k_*, *p_i,j,k_*), where *n* represents the number of detections of a species (*i*) at a location (*j*) for each replicate count (*k*) and follows a binomial distribution. We modeled the detection probability of each species in a pair based on a different set of variables expected to affect the observation process, which is detailed below.

### 2.5 Covariates

We hypothesized that these species would vary in their habitat preferences and tolerance to human disturbance, so we incorporated natural and anthropogenic covariates into our co‐abundance models. We predicted that habitat type and cover, distance to water (m), distance to the nearest human settlement (m), and human activity would influence species abundance (Ramesh et al., 2017; Rich et al., 2017; Schuette et al., 2013).

We used the Normalized Difference Vegetation Index (NDVI) calculated from a cloud‐free, Landsat 8 image (Path 67, Row 73) acquired July 2017 and downloaded from USGS Earth Explorer (https://earthexplorer.usgs.gov/) to represent habitat type, cover, and forage availability (DeFries & Townshend, 1994; Ladle, Steenweg, Shepherd, & Boyce, 2018; Pettorelli et al., 2005). We created a land cover map using a random forest classification model, our field notes, and Google Earth imagery, but based on the results of an ANOVA test and visual assessments of the two maps, we determined that NDVI values provided the same information as our land cover map. We therefore used NDVI instead of the categorical land cover map because it is a continuous variable frequently used in occupancy analyses (Burton, Sam, Balangtaa, & Brashares, 2012; Rich et al., 2017). We calculated the mean NDVI within a 500 m buffer surrounding each camera trap to determine how much each carnivore would likely use that location based on the general vegetation attributes of the nearby area (Carter et al., 2013; Ladle et al., 2018). We chose 500 m because it is the approximate size of a genet's home range, which is the smallest known home range of our three species (Estes, 2012; Williams et al., 2017).

To measure how water availability affects species abundance, we combined the GPS points we took from the ground where we followed creeks and rivers with spatial river data from the HydroSHEDS dataset (Lehner, Verdin, & Jarvis, 2006) to determine the location of permanent water sources in our study area. We then calculated the distance from each camera trap to the nearest water source in ArcGIS 10.5.1.

For our anthropogenic variables, we estimated human activity levels as the proportion of days people or vehicles were detected at each camera trap, for the number of days each trap was active. We did not believe that human activity would impact detection because these species are nocturnal (Estes, 2012), but we predicted that areas with greater human activity, such as those where logging was occurring (Brodie et al., 2018) or near an active road (Smith et al., 2018), may affect the abundance of carnivores using that area. We also included the distance of each trap to the nearest settlement in kilometers, calculated in ArcGIS 10.5.1. The abundance model is therefore specified as:

$$\log\left(\lambda_{i,j}\right)=\alpha 0_{i}+\alpha 1_{i}{\left(\text{NDVI}\right)}_{j}+\alpha 2_{i}{\left(\text{water}\right)}_{j}+\alpha 3_{i}{\left(\text{settle}\right)}_{j}+\alpha 4_{i}{\left(\text{human}\right)}_{j}+\delta_{i}\left(N_{1,j}\right).$$

We included a different set of site‐level covariates for the detection model that we expected may affect the localized detection process or space use of an animal. Carnivores often utilize trails and roads when traveling (Cusack et al., 2015; Kolowski & Forrester, 2017), so we included a binary variable for whether a trap was located on (1) or off (0) a trail. We also included a binary variable for if a trap consisted of two cameras (1) or one (0) which may affect the detectability of smaller species (Pease, Nielsen, & Holzmueller, 2016). We used the Julian date for each sampling day of each individual camera trap site to help account for changing detection rates over the study period and possible bias associated with pseudoreplication. This covariate measures the changes in detection rates that may have to do with seasonality or the progression of our sampling, with only one block of cameras active at a time and each block successively following the previous one. Finally, we calculated the slope at each camera trap using a Digital Elevation Model in ArcGIS 10.5.1 (Ahumada et al., 2013; Brodie et al., 2018; Rovero, Martin, Rosa, Ahumada, & Spitale, 2014). We therefore specified the detection model as:

$$\log\text{it}\left(p_{i,j,k}\right)=\beta 0_{i}+\beta 1_{i}{\left(\text{trail}\right)}_{j}+\beta 2_{i}{\left(\text{paired}\right)}_{j}+\beta 3_{i}{\left(\text{slope}\right)}_{j}+\beta 4_{i}{\left(\text{survey days}\right)}_{j}$$

We checked all continuous covariates for collinearity with the Pearson correlation coefficient. We initially considered including elevation in our models, but it was significantly correlated with distance from water (Pearson *r* = .71), so we discarded this covariate. Additionally, variation in detection probabilities may partially depend on which of our sampling blocks the camera traps were located in. To account for this, we developed models that incorporated a random effect for our blocks, using several combinations of the variables listed above. However, models did not converge when these random effects were included. Therefore, instead of using models with unreliable coefficient estimates, we dropped blocks as a random effect and used fixed‐effect models in subsequent analysis in order to make stronger inferences on the effects of ecologically meaningful, camera trap‐level covariates (i.e., distance of each camera trap from water, distance from human settlements, human activity levels at each camera trap, average NDVI within 500 m of each camera trap).

We used a Bayesian approach with minimally informative priors (McElreath, 2016) to estimate model parameters. This approach provides two advantages. First, Bayesian analysis allows for the explicit estimates of latent *N*_1,_ *_j_* values which are used to estimate N_2,_ *_j_* values (Brodie et al., 2018). Second, by assigning regularizing priors to all the parameter coefficients, we reduce overfitting while creating a “skeptical” model, which interprets values above or below zero to be less plausible. Therefore, we are more confident in the significance of a parameter estimate if the 95% CIs do not overlap zero (McElreath, 2016). We implemented our models with R (R Core Team, 2013) using the package R2jags (Plummer, 2011). We ran three chains of 100,000 iterations and discarded the first 50,000 as a burn‐in for each species pair and thinned the remaining 50,000 iterations by 20. We assessed model convergence by visually examining trace plots and with the Gelman–Rubin diagnostic, where Rhat values >1.1 indicate poor convergence (Gelman, Hwang, & Vehtari, 2014).

## 3 RESULTS

### 3.1 Carnivore detection data

Cameras were active for 2,090 trap days. Two of the sites had malfunctioning cameras, leaving 75 sites to analyze. We obtained 168 independent detections of bushy‐tailed mongooses at 36 of our camera traps, 152 detections of African civets at 29 traps, and 120 detections of large‐spotted genets at 25 traps. Five other carnivore species were detected at much lower frequencies: Marsh mongooses (*Atilax paludinosus*) were detected 40 times; servals (*Leptailurus serval*) were detected 12 times; leopards were detected 8 times; white‐tailed mongooses (*Ichneumia albicauda*) were detected 6 times; and honey badgers (*Mellivora capensis*) were detected 5 times.

### 3.2 Temporal partitioning

**FIGURE 3:**
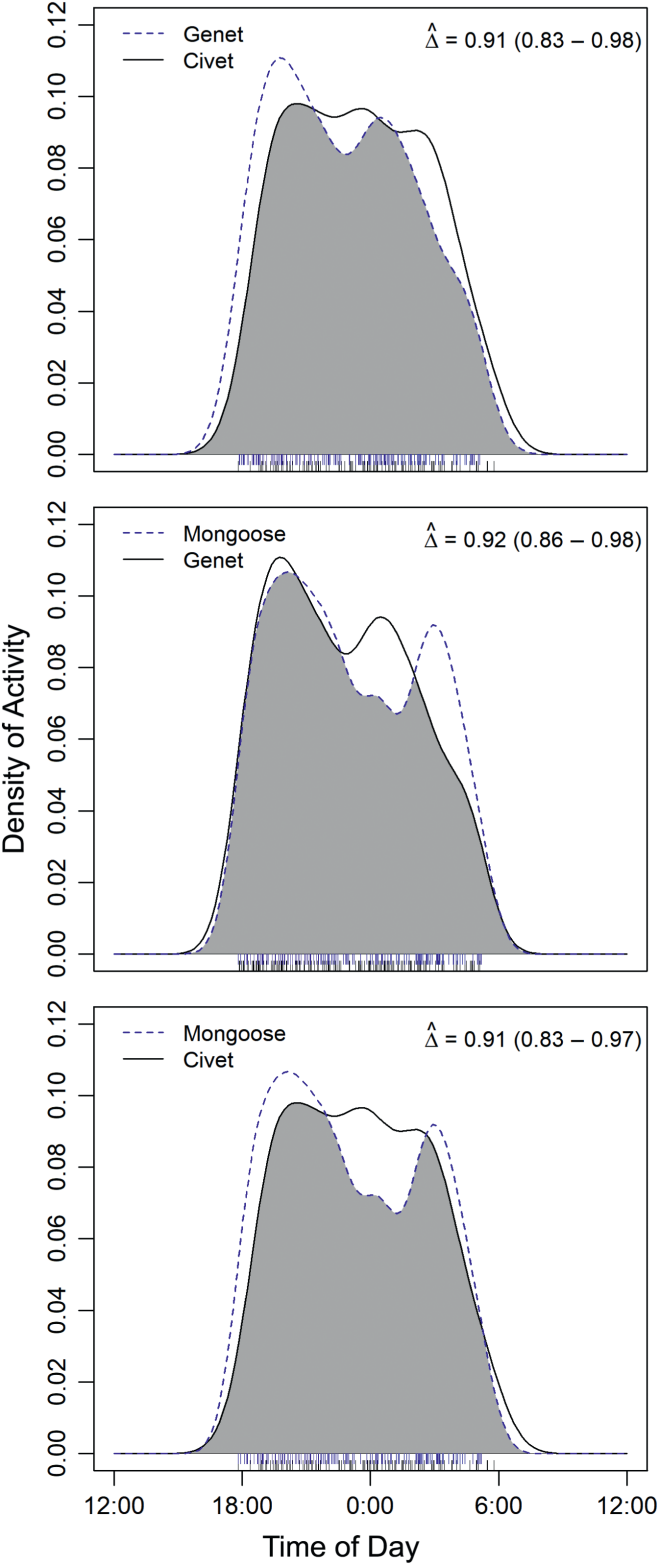
Overlap of daily activity patterns for each species pair across all detections. The estimate of overlap (Δ, with 0 indicating no overlap and 1 indicating complete overlap) is indicated by the gray area. Blue and black ticks indicate the raw time stamps used to create the density curves, and 95% confidence intervals are given in parentheses. Activity patterns of each species pair strongly overlapped (Δ > 0.8)

**FIGURE 4:**
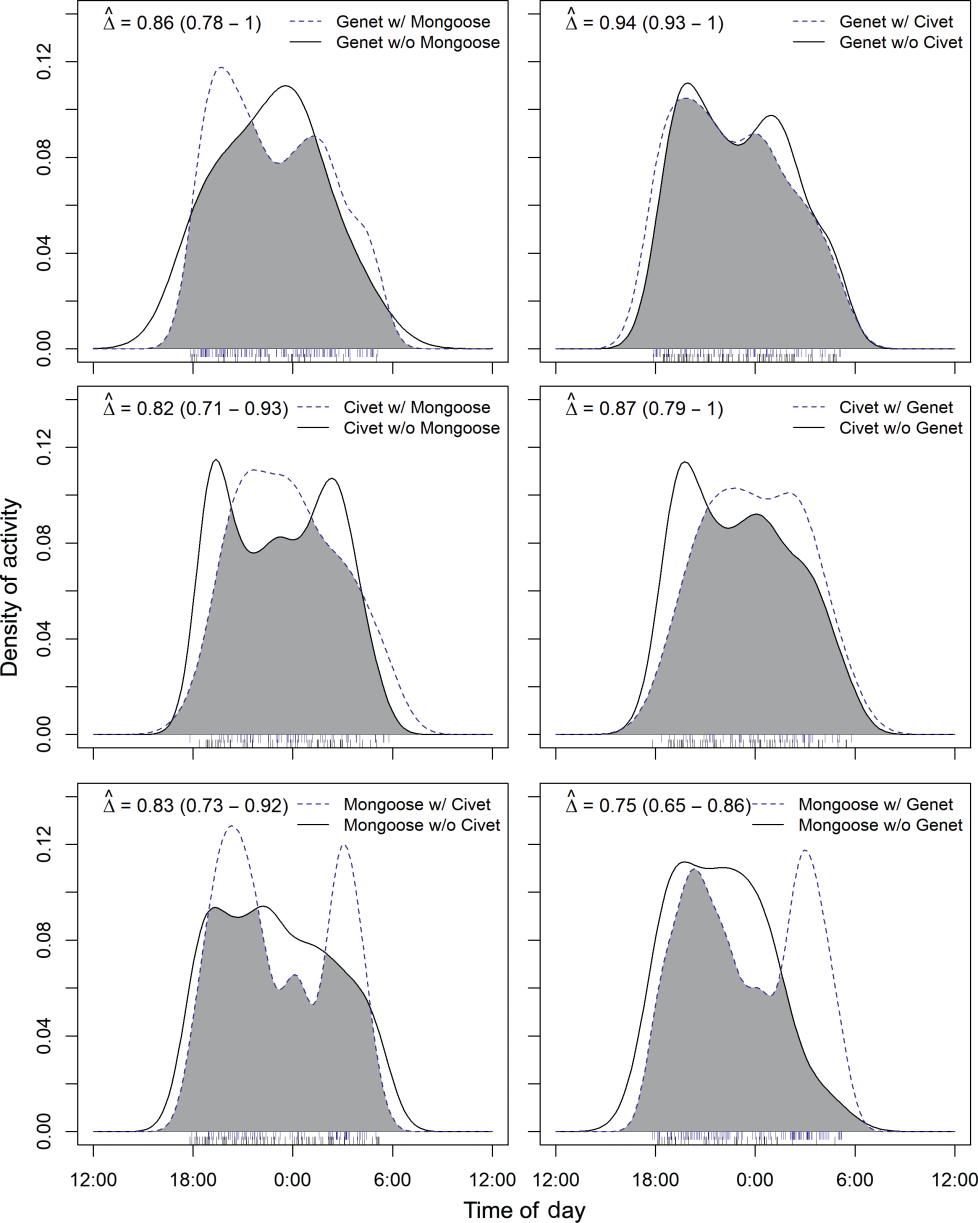
Daily activity pattern overlap for each species for when they were detected at the same camera traps as their competitors (blue dashed lines) and for when they were detected at camera traps where their competitors were not (black lines). The estimate of overlap (Δ, with 0 indicating no overlap and 1 indicating complete overlap) is indicated by the gray area. Blue and black ticks indicate the raw time stamps used to create the density curves, and 95% confidence intervals are given in parentheses. Activity patterns between genets and civets changed the least when the other was present, while mongoose activity patterns varied more greatly if civets or genets used the same area

Each of our three mesocarnivores was active between the hours of 6 p.m. and 6 a.m., and each species’ activity patterns strongly overlapped with those of their potential competitors across the study site ( $\hat{D_{1}}>0.8$ ; Figure 3). However, bushy‐tailed mongooses appear to shift to being more crepuscular when using the same site as a potential competitor (Figure 4). Mongoose activity patterns remained strongly overlapping with civet activity patterns ( $\hat{D_{1}}=0.83$ ), but did not strongly overlap with genet activity ( $\hat{D_{1}}=0.75$ ). Further, civets and mongooses appear to have inverse activity patterns when in the presence of the other (Figure 4). There was very little difference in the activity times of genets when in the presence of a competitor, however, and their activity patterns hardly changed at all when in the presence of civets ( $\hat{D_{1}}=0.94$ ; Figure 4).

### 3.3 Spatial partitioning

**FIGURE 5:**
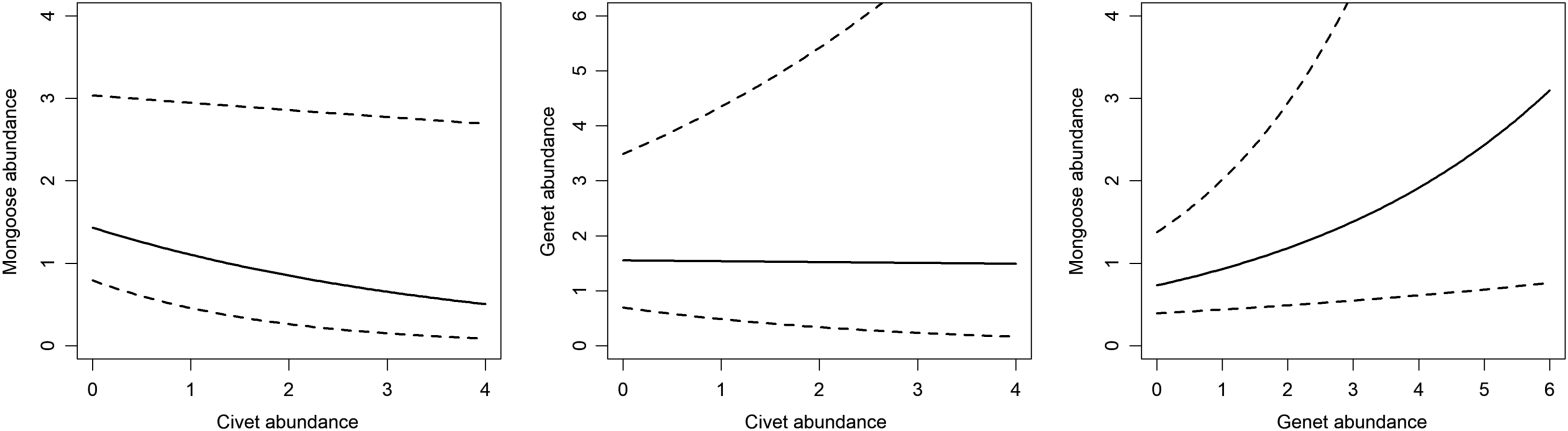
Estimated correlations between species abundances. The black lines represent the mean correlation estimate for each pair: −0.26 for bushy‐tailed mongooses and African civets, 0.24 for bushy‐tailed mongooses and large‐spotted genets, and −0.01 for large‐spotted genets and African civets (on the logit scale). Dashed lines indicate 95% confidence intervals

**FIGURE 6:**
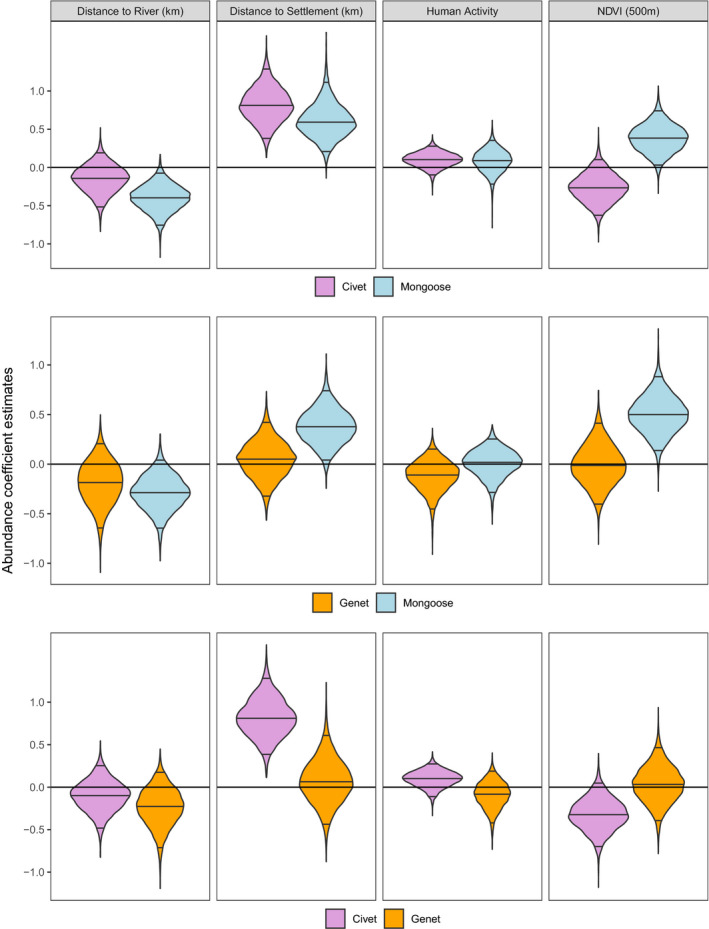
Violin plots of the coefficient estimates for each variable in the abundance models of each species pair (African civets shown in purple, bushy‐tailed mongooses shown in blue, and large‐spotted genets shown in orange). Black lines through the violins indicate estimates at the 2.5%, 50%, and 97.5% intervals. Mongooses were more likely to use sites farther away from settlements, close to rivers, and in greener, or more forested, areas. In contrast, civets’ site use was not significantly correlated with rivers or NDVI, but civets were also more likely to use sites farther away from settlements. Genet site use was not significantly correlated with any of the covariates. NDVI, Normalized Difference Vegetation Index

Our models estimated a negative correlation between African civet (largest of the mesocarnivores) and bushy‐tailed mongoose site use, a positive correlation between large‐spotted genet and bushy‐tailed mongoose site use (about the same size), and African civet and large‐spotted genet site use were not correlated (Figure 5). Civet and mongoose site use was strongly correlated with settlement proximity, and mongooses were more likely to use more forested sites (areas with high NDVI; Figure 6). Genet site use did not have a strong relationship with any of the habitat variables in the abundance models (Figure 6). The slope at each camera trap, the dates traps were active, and whether a camera trap was placed on a trail were significant predictors for these species’ detection probabilities, detailed below, but whether a camera trap consisted of a single camera or pair of cameras was not strongly correlated with any of their detection probabilities (Table 2). The estimated effect of each coefficient in the detection and abundance models and their 95% CIs shifted slightly for each species depending on which other species they were paired with, which is detailed below.

**TABLE 2:** Detection parameter estimates (on the logit scale) for African civets, bushy‐tailed mongooses, and large‐spotted genets detected in a forestry concession in central Mozambique

Species	On trail	Paired cameras	Survey days	Slope
Civet	1.82*	0.04	−0.51*	−0.11
Genet	2.87*	−0.04	0.22	−0.18
Civet	1.76*	0.04	−0.51*	−0.09
Mongoose	0.03	−0.06	−0.13	−0.44*
Genet	2.95*	−0.14	0.22	−0.18
Mongoose	−0.04	−0.20	−0.11	−0.47*

“On trail” is a binary variable for if a camera trap was placed on a road or trail. “Paired cameras” is also a binary variable for if a camera trap had one (0) or two (1) cameras. “Survey days” refers to the Julian date of each survey day, and the slope is the slope measured at each camera trap at 30 m resolution. Asterisks indicate estimates with 95% credibility intervals that do not overlap zero. Civets and genets were more likely to be detected on trails. Civets were less likely to be detected later in the dry season, and mongooses were less likely to be detected on steeper slopes.

### 3.4 Civet–genet

Civet abundance did not have a significant relationship with genet abundance (mean: −0.06, 95% CI: −0.36 to 0.22; Figure 5). In the civet–genet model, civet abundance was strongly related to distance from the nearest settlement, with abundance increasing as distance from settlements increased (mean: 0.82, 95% CI: 0.40–1.28). In contrast, there was not a strong relationship between genet abundance and settlement distance (mean: 0.07, 95% CI: −0.43 to 0.60; Figure 6). The other covariates in the abundance model (distance to rivers, NDVI, and human activity) did not have strong effects on either genet or civet abundance (Figure 6).

The detection probability of both species significantly increased for camera traps that were located on a trail. Civet detection probability also decreased further into the dry season. The slope and number of cameras at each trap did not significantly change either species’ detection probabilities (Table 2).

### 3.5 Civet–mongoose

Our models estimated a negative relationship between civet and bushy‐tailed mongoose abundance (mean: 0.26, 95% CI: −0.55 to −0.03; Figure 5). Both species’ abundances were positively related to increasing distance from settlements (civet mean: 0.82, 95% CI: 0.39–1.29; mongoose mean: 0.61, 95% CI: 0.22–1.12). Bushy‐tailed mongoose abundance was also positively correlated with NDVI (mean: 0.38, 95% CI: 0.04–0.74) and negatively correlated with distance to water (mean: −0.40, 95% CI: −0.76 to −0.08) with higher abundances predicted in forested areas near water. In contrast, civet abundance had a weaker, but negative relationship with NDVI (mean: −0.26, 95% CI: −0.62 to 0.10; Figure 6).

Civet detection probabilities were higher later in the season and when cameras were placed on trails but were not strongly related to the number of cameras or the slope at each site. Bushy‐tailed mongoose detection probabilities decreased for traps located near steeper slopes and were not strongly correlated with any of the other detection covariates (paired cameras, on/off trails, and date; Table 2).

### 3.6 Genet–mongoose

Genet and bushy‐tailed mongoose abundances were positively correlated (mean: 0.24, 95% CI: 0.11–0.38; Figure 5). None of the parameter coefficient estimates significantly differed between the two species, despite mongoose abundance being more strongly related to NDVI (mean: 0.50, 95% CI: 0.14–0.88), and distance from settlement (mean: 0.38, 95% CI: 0.04–0.74; Figure 6). Bushy‐tailed mongooses, again, were less likely to be detected at sites with steeper slopes, but neither species had significant relationships with any of the other covariates (Table 2).

## 4 DISCUSSION

Although important to ecosystem functioning, little is known about mesocarnivore ecology in human‐modified landscapes. We have provided evidence for fine‐scale spatial and temporal partitioning among sympatric carnivores in a forestry area of Mozambique. Our results indicate that after accounting for differences in habitat preferences and sensitivities to anthropogenic factors, bushy‐tailed mongooses and African civets partition in space and time. Further, while large‐spotted genet site use and activity patterns were not affected by either of the other two species, bushy‐tailed mongooses seemed to adjust their activity patterns to avoid genets. While our findings did not lend support to either of our hypotheses, the spatiotemporal patterns of these species warrant further exploration.

Differences in foraging strategies, dietary preferences, and the relative abundance of food may explain how genets can occupy the same spatial and temporal niches of these other two mesocarnivores (Angelici & Luiselli, 2005; Caro & Stoner, 2003; Estes, 2012; Ray & Sunquist, 2001; Waser, 1980). Genets are more arboreal than civets and mongooses, which may allow for an even finer scale spatial partition between these species (Maddock & Perrin, 1993). However, the high spatial overlap of mongoose and genet site use is likely further facilitated by mongooses avoiding genets in time (Figure 4). Little is known about the foraging behaviors of bushy‐tailed mongooses, but genets are more carnivorous than civets; they often stalk and hunt prey whereas civets are ambush carnivores and more opportunistic omnivores (Estes, 2012; Ray & Sunquist, 2001). Such differences have been shown to mediate competitive exclusion in other systems, such as the avoidance of Iberian lynx (*Lynx pardinus*) by red foxes but not by Eurasian badgers (*Meles meles*), which have a more distinctive foraging strategy (Fedriani, Palomares, & Delibes, 1999). Finally, shared resources tracked by all these species, such as prey species (i.e., rats, *Cricetomys gambianus*, *Thryonomys gregorianus*) may be abundant, as suggested by our camera trapping detections but not explicitly quantified. If abundant resources can support a higher number of these mesocarnivores, competitive interactions or resource partitioning would not be necessary (Brodie et al., 2018). Our study did not quantify forage or prey availability since our study species consume such a variety of animal and plant species (Caro & Stoner, 2003; Williams et al., 2017), but Rich et al. (2017) found that, generally, carnivore occupancy in Botswana depended more on resource availability than the presence of competing species. Indeed, civet occupancy was negatively related to the detection rates of similarly sized carnivores in Botswana during the dry season, but positively related to them in the wet season, possibly due to greater resource availability (Rich et al., 2017).

Civets and mongooses appeared to avoid each other in space and time at fine scales at our site (Figures 4 and 5). In contrast to genets and mongooses, though, civets and mongooses appear to both adjust their activity patterns when using the same sites, exhibiting inverse activity patterns with and without the other present, rather than one more strongly avoiding the other. However, civets and mongooses may be seeking different resources that were not represented in our models, indicating that the negative relationship between mongoose and civet site use more accurately represents different preferences rather than competition or avoidance. For example, civets are more frugivorous than others in their guild and have been considered hypocarnivorous (less than 30% of its diet consists of meat, Amiard, Kruger, Mullers, & Schipper, 2015; Ray & Sunquist, 2001; Waser, 1980). Civets are also typically seen in more open habitats, whereas bushy‐tailed mongooses have exhibited strong avoidance of open habitats (Pettorelli et al., 2010). However, this variance should be captured in the NDVI variable of our models, which does show positive correlations between mongoose site use and forested areas and the opposite (though weaker) relationship with civet site use.

It is important to understand intraguild interactions among mesocarnivores in multi‐use landscapes, where the presence of people may drive different patterns than what would be expected in protected areas (Massara, Paschoal, Bailey, Doherty, & Chiarello, 2016; Schuette et al., 2013; Waser, 1980). Civets and mongooses were both more likely to occur in higher numbers farther away from human settlements. Other studies have documented similar patterns, where carnivore occupancy is reduced near permanent settlements (Burton et al., 2012; Carter et al., 2013; Schuette et al., 2013; Williams et al., 2017). This result is disconcerting because mesocarnivores provide ecosystem services, from which people could benefit. For example, these species likely play a large part in limiting rodent and other pest populations in cropland areas, and, by extension, limiting the spread of zoonotic diseases (Ostfeld & Holt, 2004; Williams et al., 2017). Additionally, civets (e.g., *Paguma larvata*, *Paradoxurus hermaphroditus*, *Viverra zibetha*) in particular are considered important seed dispersers (Caughlin et al., 2014; Nakashima, Inoue, Inoue‐Murayama, & Abd. Sukor JR., 2010). However, these services are reliant on both the abundance and diversity of mesocarnivores, which, as supported by our results as well as other studies, can be limited in human‐altered areas (Burton et al., 2012; Schuette et al., 2013; Williams et al., 2017). The selection of habitats farther from settlements by both mongooses and civets likely further limits resource availability and opportunities for niche partitioning (Massara et al., 2016; Moll et al., 2018). Human populations are projected to rapidly grow in Mozambique, including in the Gorongosa region (United Nations, 2017). The expansion of settlements may exacerbate the negative interactions between civets and mongooses by pushing them out of viable habitats and facilitating more interactions between these and other potentially competing species whose interactions and basic ecologies remain unknown (Do Linh San et al., 2013).

Competitive interactions are also important to consider for conservation planning, specifically the restoration of large carnivore populations. Mesocarnivores often spatially or temporally avoid large carnivores to reduce the potential for competition or even predation (Johnson & VanDerWal, 2009). Leopards are the only known large carnivore to occur at our site, and they were rarely detected, likely due to low population sizes following Mozambique's civil war (Bouley et al., 2018; Easter et al., 2019). The recovery of leopards and other large carnivores to prewar densities and facilitation of their movement between protected areas in the region, including through our study site, is a priority for Gorongosa National Park managers. We were unable to test how leopards affected mesocarnivore abundance due to low sample sizes, but their presence and recovery could alter intraguild dynamics. For example, in one of the few studies that examined the effect of leopards on mesocarnivore occupancy, Ramesh et al. (2017) found that honey badgers (*Mellivora capensis*), slender mongooses (*Galerella sanguinea*), and striped polecats (*Ictonyx striatus*) were detected less often at sites where leopards were detected. Additionally, leopards have been known to kill and eat civets (Palomares & Caro, 1999). Leopards may therefore reduce the amount of available habitat for subordinate carnivores. Alternatively, they may suppress medium‐sized carnivores such as civets, releasing mongooses, other competitors, and their prey from interference and predatory pressures. These carnivore cascades have been noted in North America, Australia, Europe, and East Africa (e.g., Creel & Creel, 1996; Johnson & VanDerWal, 2009; Levi & Wilmers, 2012; Pasanen‐Mortensen et al., 2013; Sivy et al., 2017).

These multifaceted interactions between carnivores, people, and their environment are critical to understanding the spatiotemporal dynamics of sympatric carnivores. Our study demonstrates the importance of considering each of these elements to better understand the ecology of these mesocarnivores, which few studies have addressed. However, the following limitations of our study in addition to the other possible drivers of spatiotemporal partitioning discussed above (e.g., prey and forage availability) should be carefully considered. First, as discussed in the methods, we experienced issues with overparameterization which limited our ability to fully account for the possible effects of pseudoreplication between our camera trap blocks or individual sites. Second, African civets have larger home ranges (see Table 1) than the distance between our camera traps (~1.5 km), and their detections may be autocorrelated. Lastly, some basic ecological information is still lacking for these species, such as the foraging strategies, home range sizes, and habitat preferences of bushy‐tailed mongooses, which would help untangle their relationships with sympatric species. Such studies are much needed in anthropogenic landscapes, which are ubiquitous worldwide.

## AUTHOR CONTRIBUTION

**Tara Easter**: Conceptualization (lead); Data curation (lead); Formal analysis (lead); Funding acquisition (equal); Investigation (lead); Methodology (lead); Project administration (equal); Visualization (lead); Writing‐original draft (lead); Writing‐review & editing (lead). **Paola Bouley**: Conceptualization (supporting); Data curation (supporting); Funding acquisition (equal); Investigation (supporting); Project administration (supporting); Writing‐review & editing (supporting). **Neil Carter**: Conceptualization (supporting); Funding acquisition (equal); Project administration (equal); Writing‐original draft (supporting); Writing‐review & editing (equal).

## Data Availability

All data and code are publicly available on Dryad: https://doi.org/10.5061/dryad.612jm640s.

## References

[ece36290-bib-0001] Admasu, E. , Thirgood, S. J. , Bele, A. , & Laurenson, M. K. (2004). A note on the spatial ecology of African civet *Civettictis civetta* and common genet *Genetta genetta* in farmland in the Ethiopian Highlands. African Journal of Ecology, 42, 160–162. 10.1111/j.1365-2028.2004.00496.x

[ece36290-bib-0002] Ahumada, J. A. , Hurtado, J. , & Lizcano, D. (2013). Monitoring the Status and Trends of Tropical Forest Terrestrial Vertebrate Communities from Camera Trap Data: A Tool for Conservation. PLoS ONE, 8, 6–9. 10.1371/journal.pone.0073707 PMC3762718 24023898

[ece36290-bib-0003] Allen, M. L. , Peterson, B. , & Krofel, M. (2018). No respect for apex carnivores: Distribution and activity patterns of honey badgers in the Serengeti. Mammalian Biology, 89, 90–94. 10.1016/j.mambio.2018.01.001

[ece36290-bib-0004] Amiard, P. J. , Kruger, C. V. , Mullers, R. H. E. , & Schipper, J. (2015). The diet of African Civet *Civettictis civetta* in two vegetation types of the Savannah biome in South Africa. Small Carnivore Conservation, 52 & 53, 4–12.

[ece36290-bib-0005] Angelici, F. M. , & Luiselli, L. (2005). Habitat associations and dietary relationships between two genets, *Genetta maculata* and *Genetta cristata* . Revue D Ecologie, 60, 17370993.

[ece36290-bib-0006] Athreya, V. , Odden, M. , Linnell, J. D. C. , Krishnaswamy, J. , & Karanth, U. (2013). Big cats in our backyards: Persistence of large carnivores in a human dominated landscape in India. PLoS ONE, 8, 2–9. 10.1371/journal.pone.0057872 PMC3590292 23483933

[ece36290-bib-0007] Berger, J. (2007). Fear, human shields and the redistribution of prey and predators in protected areas. Biology Letters, 3, 620–623. 10.1098/rsbl.2007.0415 17925272 PMC2391231

[ece36290-bib-0008] Bouley, P. , Poulos, M. , Branco, R. , & Carter, N. H. (2018). Post‐war recovery of the African lion in response to large‐scale ecosystem restoration. Biological Conservation, 227, 233–242. 10.1016/j.biocon.2018.08.024

[ece36290-bib-0009] Brodie, J. F. , Helmy, O. E. , Mohd‐Azlan, J. , Granados, A. , Bernard, H. , Giordano, A. J. , & Zipkin, E. (2018). Models for assessing local‐scale co‐abundance of animal species while accounting for differential detectability and varied responses to the environment. Biotropica, 50, 5–15. 10.1111/btp.12500

[ece36290-bib-0010] Burton, A. C. , Sam, M. K. , Balangtaa, C. , & Brashares, J. S. (2012). Hierarchical multi‐species modeling of carnivore responses to hunting, habitat and prey in a West African protected area. PLoS ONE, 7, e38007 10.1371/journal.pone.0038007 22666433 PMC3364199

[ece36290-bib-0011] Cardillo, M. , Mace, G. M. , Jones, K. E. , Bielby, J. , Bininda‐Emonds, O. R. P. , Sechrest, W. , … Purvis, A. (2005). Multiple causes of high extinction risk in large mammal species. Science, 309, 1239–1241. 10.1126/science.1116030 16037416

[ece36290-bib-0012] Caro, T. M. , & Stoner, C. J. (2003). The potential for interspecific competition among African carnivores. Biological Conservation, 110, 67–75. 10.1016/S0006-3207(02)00177-5

[ece36290-bib-0013] Carter, N. H. , Gurung, B. , Viña, A. , Campa, H. III , Karki, J. B. , & Liu, J. (2013). Assessing spatiotemporal changes in tiger habitat across different land management regimes. Ecosphere, 4, 1–19. 10.1890/ES13-00191.1

[ece36290-bib-0014] Carter, N. , Jasny, M. , Gurung, B. , & Liu, J. (2015). Impacts of people and tigers on leopard spatiotemporal activity patterns in a global biodiversity hotspot. Global Ecology and Conservation, 3, 149–162. 10.1016/j.gecco.2014.11.013

[ece36290-bib-0015] Carter, N. H. , Shrestha, B. K. , Karki, J. B. , Pradhan, N. M. B. , & Liu, J. (2012). Coexistence between wildlife and humans at fine spatial scales. Proceedings of the National Academy of Sciences, 109, 15360–15365. 10.1073/pnas.1210490109 PMC3458348 22949642

[ece36290-bib-0016] Caughlin, T. T. , Ferguson, J. M. , Lichstein, J. W. , Zuidema, P. A. , Bunyavejchewin, S. , & Levey, D. J. (2014). Loss of animal seed dispersal increases extinction risk in a tropical tree species due to pervasive negative density dependence across life stages. Proceedings of the Royal Society B: Biological Sciences, 282, 20142095 10.1098/rspb.2014.2095 PMC4262173 25392471

[ece36290-bib-0017] Creel, S. , & Creel, N. M. (1996). Limitation of African wild dogs by competition with larger carnivores. Conservation Biology, 10, 526–538. 10.1046/j.1523-1739.1996.10020526.x

[ece36290-bib-0018] Crooks, K. R. , & Soulé, M. E. (1999). Mesopredator release and avifaunal extinctions in a fragmented system. Nature, 400, 563–566. 10.1038/23028

[ece36290-bib-0019] Cusack, J. J. , Dickman, A. J. , Rowcliffe, J. M. , Carbone, C. , Macdonald, D. W. , & Coulson, T. (2015). Random versus game trail‐based camera trap placement strategy for monitoring terrestrial mammal communities. PLoS ONE, 10, e0126373 10.1371/journal.pone.0126373 25950183 PMC4423779

[ece36290-bib-0020] de Satgé, J. , Teichman, K. , & Cristescu, B. (2017). Competition and coexistence in a small carnivore guild. Oecologia, 184, 873–884. 10.1007/s00442-017-3916-2 28733835

[ece36290-bib-0021] DeFries, R. S. , & Townshend, J. R. G. (1994). NDVI‐derived land cover classifications at a global scale. International Journal of Remote Sensing, 15, 3567–3586. 10.1080/01431169408954345

[ece36290-bib-0022] Do Linh San, E. , Ferguson, A. W. , Belant, J. L. , Schipper, J. , Hoffman, M. , Gaubert, P. , … Somers, M. J. (2013). Conservation status, distribution and species richness of small carnivores in Africa. Small Carnivore Conservation, 48, 4–18.

[ece36290-bib-0023] Donadio, E. , & Buskirk, S. W. (2006). Diet, morphology, and interspecific killing in Carnivora. The American Naturalist, 167, 524–536. 10.1086/501033 16670995

[ece36290-bib-0024] Durant, S. M. (1998). Competition refuges and coexistence: An example from Serengeti carnivores. Journal of Animal Ecology, 67, 370–386. 10.1046/j.1365-2656.1998.00202.x

[ece36290-bib-0025] Easter, T. , Bouley, P. , & Carter, N. (2019). Opportunities for biodiversity conservation outside of Gorongosa National Park, Mozambique: A multispecies approach. Biological Conservation, 232, 217–227. 10.1016/j.biocon.2019.02.007

[ece36290-bib-0026] Ellis, E. C. (2011). Anthropogenic transformation of the terrestrial biosphere. Philosophical transactions. Series A: Mathematical, Physical, and Engineering Sciences, 369, 1010–1035. 10.1098/rsta.2010.0331 21282158

[ece36290-bib-0027] Estes, R. D. (2012). The behavior guide to African mammals: including hoofed mammals, carnivores, and primates. 20th Anniv. Berkeley and Los Angeles, California, and London, England: University of California Press.

[ece36290-bib-0028] Fedriani, J. M. , Fuller, T. K. , Sauvajot, R. M. , & York, E. C. (2000). Competition and intraguild predation among three sympatric carnivores. Oecologia, 125, 258–270. 10.1007/s004420000448 24595837

[ece36290-bib-0029] Fedriani, J. M. , Palomares, F. , & Delibes, M. (1999). Niche relations among three sympatric Mediterranean carnivores. Oecologia, 121, 138–148. 10.1007/s004420050915 28307883

[ece36290-bib-0030] Fuller, T. K. , Biknevicius, A. R. , & Kat, P. W. (1990). Movements and behavior of large spotted genets (*Genetta maculata* Gray 1830) near Elmenteita Kenya (Mammalia Viverridae). Tropical Zoology, 3, 13–19. 10.1080/03946975.1990.10539446

[ece36290-bib-0031] Gaynor, K. M. , Brown, J. S. , Middleton, A. D. , Power, M. E. , & Brashares, J. S. (2019). Landscapes of fear: Spatial patterns of risk perception and response. Trends in Ecology and Evolution, 34, 355–368. 10.1016/j.tree.2019.01.004 30745252

[ece36290-bib-0032] Gelman, A. , Hwang, J. , & Vehtari, A. (2014). Understanding predictive information criteria for Bayesian models. Statistics and Computing, 24, 997–1016. 10.1007/s11222-013-9416-2

[ece36290-bib-0033] Harrison, D. J. , Bissonette, J. A. , & Sherburne, J. A. (1989). Spatial relationships between coyotes and red foxes in Eastern Maine. The Journal of Wildlife Management, 53, 181 10.2307/3801327

[ece36290-bib-0034] Johnson, C. N. , & VanDerWal, J. (2009). Evidence that dingoes limit abundance of a mesopredator in eastern Australian forests. Journal of Applied Ecology, 46, 641–646. 10.1111/j.1365-2664.2009.01650.x

[ece36290-bib-0035] Kiffner, C. , Wenner, C. , LaViolet, A. , Yeh, K. , & Kioko, J. (2015). From savannah to farmland: Effects of land‐use on mammal communities in the Tarangire‐Manyara ecosystem, Tanzania. African Journal of Ecology, 53, 156–166. 10.1111/aje.12160

[ece36290-bib-0036] Kingdon, J. (2015). The Kingdon field guide to African mammals. London, UK: Bloomsbury Publishing.

[ece36290-bib-0037] Kolowski, J. M. , & Forrester, T. D. (2017). Camera trap placement and the potential for bias due to trails and other features. PLoS ONE, 12, e0186679 10.1371/journal.pone.0186679 29045478 PMC5646845

[ece36290-bib-0038] Ladle, A. , Steenweg, R. , Shepherd, B. , & Boyce, M. S. (2018). The role of human outdoor recreation in shaping patterns of grizzly bear‐black bear co‐occurrence. PLoS ONE, 13, 1–16. 10.1371/journal.pone.0191730 PMC5794087 29389939

[ece36290-bib-0039] Lehner, B. , Verdin, K. , & Jarvis, A. (2006). HydroSHEDS technical documentation. US, Washington, DC: World Wildlife Fund Retrieved from http://hydrosheds.cr.usgs.gov

[ece36290-bib-0040] Levi, T. , & Wilmers, C. (2012). Wolves – coyotes – foxes : A cascade among carnivores. Ecology, 93, 921–929. 10.1890/11-0165.1 22690642

[ece36290-bib-0041] MacKenzie, D. I. , Nichols, J. D. , Lachman, G. B. , Droege, S. , Royle, A. A. , & Langtimm, C. A. (2002). Estimating site occupancy rates when detection probabilities are less than one. Ecology, 83, 2248–2255. 10.1890/0012-9658(2002)083[2248:ESORWD]2.0.CO;2

[ece36290-bib-0042] Mackenzie, D. I. , & Royle, J. A. (2005). Designing occupancy studies: General advice and allocating survey effort. Journal of Applied Ecology, 42, 1105–1114. 10.1111/j.1365-2664.2005.01098.x

[ece36290-bib-0043] Maddock, A. H. , & Perrin, M. R. (1993). Spatial and temporal ecology of an assemblage of viverrids in Natal, South Africa. Journal of Zoology, 229, 277–287. 10.1111/j.1469-7998.1993.tb02636.x

[ece36290-bib-0044] Massara, R. L. , Paschoal, A. M. O. , Bailey, L. L. , Doherty, P. F. , & Chiarello, A. G. (2016). Ecological interactions between ocelots and sympatric mesocarnivores in protected areas of the Atlantic Forest, southeastern Brazil. Journal of Mammalogy, 97, 1634–1644. 10.1093/jmammal/gyw129

[ece36290-bib-0045] McElreath, R. (2016). Statistical rethinking: A Bayesian course with examples in R and Stan. Boca Raton, FL: CRC Press.

[ece36290-bib-0046] Meredith, M. , & Ridout, M. (2017). Overview of the overlap package. R project.1–9.

[ece36290-bib-0047] Moll, R. J. , Cepek, J. D. , Lorch, P. D. , Dennis, P. M. , Robison, T. , Millspaugh, J. J. , & Montgomery, R. A. (2018). Humans and urban development mediate the sympatry of competing carnivores. Urban Ecosystems, 21, 1–14. 10.1007/s11252-018-0758-6

[ece36290-bib-0048] Nakashima, Y. , Inoue, E. , Inoue‐Murayama, M. , & Abd. Sukor, J. R. (2010). Functional uniqueness of a small carnivore as seed dispersal agents: A case study of the common palm civets in the Tabin Wildlife Reserve, Sabah, Malaysia. Oecologia, 164, 721–730. 10.1007/s00442-010-1714-1 20602116

[ece36290-bib-0049] O’Connor, K. M. , Nathan, L. R. , Liberati, M. R. , Tingley, M. W. , Vokoun, J. C. , & Rittenhouse, T. A. G. (2017). Camera trap arrays improve detection probability of wildlife: Investigating study design considerations using an empirical dataset. PLoS ONE, 12, 1–12. 10.1371/journal.pone.0175684 PMC5396891 28422973

[ece36290-bib-0050] Oriol‐Cotterill, A. , Macdonald, D. W. , Valeix, M. , Ekwanga, S. , & Frank, L. G. (2015). Spatiotemporal patterns of lion space use in a human‐dominated landscape. Animal Behaviour, 101, 27–39. 10.1016/j.anbehav.2014.11.020

[ece36290-bib-0051] Ostfeld, R. S. , & Holt, R. D. (2004). Are predators good for your health? Evaluating evidence for top‐down regulation of zoonotic disease reservoirs. Frontiers in Ecology and the Environment, 2, 13–20. 10.1890/1540-9295(2004)002[0013:apgfyh]2.0.co;2

[ece36290-bib-0052] Palomares, F. , & Caro, T. M. (1999). Interspecific killing among mammalian carnivores. The American Naturalist, 153, 492–508. 10.1086/303189 29578790

[ece36290-bib-0053] Pasanen‐Mortensen, M. , Pyykönen, M. , & Elmhagen, B. (2013). Where lynx prevail, foxes will fail ‐ Limitation of a mesopredator in Eurasia. Global Ecology and Biogeography, 22, 868–877. 10.1111/geb.12051

[ece36290-bib-0054] Pease, B. S. , Nielsen, C. K. , & Holzmueller, E. J. (2016). Single‐camera trap survey designs miss detections: Impacts on estimates of occupancy and community metrics. PLoS ONE, 11, e0166689 10.1371/journal.pone.0166689 27902733 PMC5130212

[ece36290-bib-0055] Pettorelli, N. , Lobora, A. L. , Msuha, M. J. , Foley, C. , & Durant, S. M. (2010). Carnivore biodiversity in Tanzania: Revealing the distribution patterns of secretive mammals using camera traps. Animal Conservation, 13, 131–139. 10.1111/j.1469-1795.2009.00309.x

[ece36290-bib-0056] Pettorelli, N. , Vik, J. O. , Mysterud, A. , Gaillard, J.‐M. , Tucker, C. J. , & Stenseth, N. C. (2005). Using the satellite‐derived NDVI to assess ecological responses to environmental change. Trends in Ecology & Evolution, 20, 503–510. 10.1016/j.tree.2005.05.011 16701427

[ece36290-bib-0057] Plummer, M. (2011). JAGS: A program for the statistical analysis of Bayesian hierarchical models by Markov Chain Monte Carlo. Retrieved from http://sourceforge.net/projects/mcmc‐jags/

[ece36290-bib-0058] R Core Team . (2013). R: A language and environment for statistical computing. Vienna, Austria: R Foundation for Statistical Computing.

[ece36290-bib-0059] Ramesh, T. , & Downs, C. T. (2014). Modelling large spotted genet (*Genetta tigrina*) and slender mongoose (*Galerella sanguinea*) occupancy in a heterogeneous landscape of South Africa. Mammalian Biology, 79, 331–337. 10.1016/j.mambio.2014.05.001

[ece36290-bib-0060] Ramesh, T. , Kalle, R. , & Downs, C. T. (2017). Staying safe from top predators: Patterns of co‐occurrence and inter‐predator interactions. Behavioral Ecology and Sociobiology, 71, 41 10.1007/s00265-017-2271-y

[ece36290-bib-0061] Ray, J. C. , & Sunquist, M. E. (2001). Trophic relations in a community of African rainforest carnivores. Oecologia, 127, 395–408. 10.1007/s004420000604 28547110

[ece36290-bib-0062] Rich, L. N. , Miller, D. A. W. , Robinson, H. S. , McNutt, J. W. , & Kelly, M. J. (2017). Carnivore distributions in Botswana are shaped by resource availability and intraguild species. Journal of Zoology, 303, 90–98. 10.1111/jzo.12470

[ece36290-bib-0063] Ripple, W. J. , Estes, J. A. , Beschta, R. L. , Wilmers, C. C. , Ritchie, E. G. , Hebblewhite, M. , … Wirsing, A. J. (2014). Status and ecological effects of the world’s largest carnivores. Science, 343, 1241484 10.1126/science.1241484 24408439

[ece36290-bib-0064] Ritchie, E. G. , & Johnson, C. N. (2009). Predator interactions, mesopredator release and biodiversity conservation. Ecology Letters, 12, 982–998. 10.1111/j.1461-0248.2009.01347.x 19614756

[ece36290-bib-0065] Roemer, G. W. , Gompper, M. E. , & Van Valkenburgh, B. (2009). The ecological role of the mammalian mesocarnivore. BioScience, 59, 165–173. 10.1525/bio.2009.59.2.9

[ece36290-bib-0066] Rosenblatt, E. , Creel, S. , Becker, M. S. , Merkle, J. , Mwape, H. , Schuette, P. , & Simpamba, T. (2016). Effects of a protection gradient on carnivore density and survival: An example with leopards in the Luangwa valley, Zambia. Ecology and Evolution, 6, 3772–3785. 10.1002/ece3.2155 27231529 PMC4864144

[ece36290-bib-0067] Rota, C. T. , Ferreira, M. A. R. , Kays, R. W. , Forrester, T. D. , Kalies, E. L. , McShea, W. J. , … Millspaugh, J. J. (2016). A multispecies occupancy model for two or more interacting species. Methods in Ecology and Evolution, 7, 1164–1173. 10.1111/2041-210X.12587

[ece36290-bib-0068] Rovero, F. , Martin, E. , Rosa, M. , Ahumada, J. A. , & Spitale, D. (2014). Estimating species richness and modelling habitat preferences of tropical forest mammals from camera trap data. PLoS ONE, 9, e103300 10.1371/journal.pone.0103300 25054806 PMC4108438

[ece36290-bib-0069] Rovero, F. , Owen, N. , Jones, T. , Canteri, E. , Iemma, A. , & Tattoni, C. (2017). Camera trapping surveys of forest mammal communities in the Eastern Arc Mountains reveal generalized habitat and human disturbance responses. Biodiversity and Conservation, 26(5), 1103–1119. 10.1007/s10531-016-1288-2

[ece36290-bib-0070] Royle, J. A. (2004). N‐mixture models for estimating population size from spatially replicated counts. Biometrics, 60, 108–115. 10.1111/j.0006-341x.2004.00142.x 15032780

[ece36290-bib-0071] Schuette, P. , Wagner, A. P. , Wagner, M. E. , & Creel, S. (2013). Occupancy patterns and niche partitioning within a diverse carnivore community exposed to anthropogenic pressures. Biological Conservation, 158, 301–312. 10.1016/j.biocon.2012.08.008

[ece36290-bib-0072] Sivy, K. J. , Pozzanghera, C. B. , Grace, J. B. , & Prugh, L. R. (2017). Fatal attraction? Intraguild facilitation and suppression among predators. The American Naturalist, 190, 663–679. 10.1086/693996 29053355

[ece36290-bib-0073] Smith, J. A. , Thomas, A. C. , Levi, T. , Wang, Y. , & Wilmers, C. C. (2018). Human activity reduces niche partitioning among three widespread mesocarnivores. Oikos, 127, 890–901. 10.1111/oik.04592

[ece36290-bib-0074] Sollmann, R. , Gardner, B. , & Belant, J. L. (2012). How does spatial study design influence density estimates from spatial capture‐recapture models? PLoS ONE, 7, 1–8. 10.1371/journal.pone.0034575 PMC3335117 22539949

[ece36290-bib-0075] Stalmans, M. , & Beilfuss, R. (2008). Landscapes of Gorongosa. Sofala Province, Mozambique: National Park Available from: https://www.gorongosa.org/our‐story/science/reports/landscapes‐gorongosa‐national‐park

[ece36290-bib-0076] Thompson, C. M. , & Gese, E. M. (2007). Food webs and intraguild predation: Community interactions of a native mesocarnivore. Ecology, 88, 334–346. 10.1890/0012-9658(2007)88[334:FWAIPC]2.0.CO;2 17479752

[ece36290-bib-0077] United Nations . (2017). World Population Prospects: The 2017 Revision, Key Findings and Advance Tables. Working Paper No. ESA/P/WP/248. Retrieved from https://esa.un.org/unpd/wpp/Publications/Files/WPP2017_KeyFindings.pdf

[ece36290-bib-0078] Wang, Y. , Allen, M. L. , & Wilmers, C. C. (2015). Mesopredator spatial and temporal responses to large predators and human development in the Santa Cruz Mountains of California. Biological Conservation, 190, 23–33. 10.1016/j.biocon.2015.05.007

[ece36290-bib-0079] Waser, P. M. (1980). Small nocturnal carnivores: Ecological studies in the Serengeti. African Journal of Ecology, 18, 167–185. 10.1111/j.1365-2028.1980.tb00640.x

[ece36290-bib-0080] Wegge, P. , Odden, M. , Pokharel, C. P. , & Storaas, T. (2009). Predator‐prey relationships and responses of ungulates and their predators to the establishment of protected areas: A case study of tigers, leopards and their prey in Bardia National Park, Nepal. Biological Conservation, 142, 189–202. 10.1016/j.biocon.2008.10.020

[ece36290-bib-0081] Williams, S. T. , Maree, N. , Taylor, P. , Belmain, S. R. , Keith, M. , & Swanepoel, L. H. (2017). Predation by small mammalian carnivores in rural agro‐ecosystems: An undervalued ecosystem service? Ecosystem Services, 30, 362–371. 10.1016/j.ecoser.2017.12.006

